# Intradialytic Central Venous Oxygen Saturation is Associated with Clinical Outcomes in Hemodialysis Patients

**DOI:** 10.1038/s41598-017-09233-x

**Published:** 2017-08-17

**Authors:** Lili Chan, Hanjie Zhang, Anna Meyring-Wösten, Israel Campos, Doris Fuertinger, Stephan Thijssen, Peter Kotanko

**Affiliations:** 1 Icahn School of Medicine at Mount Sinai, New York, NY USA; 2 Renal Research Institute, New York, NY USA

## Abstract

Central venous oxygen saturation (ScvO_2_) in the superior vena cava is predominantly determined by cardiac output, arterial oxygen content, and oxygen consumption by the upper body. While abnormal ScvO_2_ levels are associated with morbidity and mortality in non-uremic populations, ScvO_2_ has received little attention in hemodialysis patients. From 1/2012 to 8/2015, 232 chronic hemodialysis patients with central venous catheters as vascular access had their ScvO_2_ monitored during a 6-month baseline period and followed for up to 36 months. Patients were stratified into upper and lower two tertiles by a ScvO_2_ of 61.1%. Survival analysis employed Kaplan-Meier curves and adjusted Cox proportional hazards models. Patients in the lower tertiles of ScvO_2_ were older, had longer hemodialysis vintage, lower systolic blood pressure, lower ultrafiltration rates, higher leukocyte counts and neutrophil-to-lymphocyte ratios. Kaplan-Meier analysis indicated a shorter survival time in the lower tertiles of ScvO_2_ (P = 0.005, log-rank test). In adjusted Cox analysis, a 1 percent point decrease in mean ScvO_2_ was associated with a 4% increase in mortality (HR 1.04 [95% CI 1.01–1.08], P = 0.044), indicating that low﻿ ScvO_2_ is associated with poor outcomes. Research on the relative contributions of cardiac output and other factors is warranted to further elucidate the pathophysiology underlying this novel finding.

## Introduction

The mortality rate of hemodialysis (HD) patients is elevated compared to the normal population^1^. The primary cause of mortality is cardiovascular disease (CVD), and there is evidence that the mechanism for CVD in HD patients differ from the traditional CVD risk factors in the general population^2, 3^. High ultrafiltration rates (UFR), episodes of intradialytic hypotension, presence of congestive heart failure (CHF) and left ventricular hypertrophy (LVH) are some of the factors that have been associated with increased mortality^4, 5^. Additionally nocturnal hypoxemia in HD patients has been demonstrated to be associated with worse cardiovascular outcomes^6, 7^.

Mixed venous oxygen saturation (SmvO_2_) and central venous oxygen saturation (ScvO2) have been used in critical care to guide fluid resuscitation^8^. SmvO_2_ is the oxygen saturation in the pulmonary artery, which receives blood from the superior vena cava, the inferior vena cava, and the coronary sinus, and therefore reflects – in the absence of arterial venous shunts – the aggregated effects of oxygen delivery to and utilization by the entire body. ScvO_2_ from upper body central venous catheters (CVC) is the oxygen saturation of blood in the superior vena cava, which reflects the aggregate of oxygen delivery to and utilization by the upper body. Although resting SmvO_2_ and ScvO_2_ differ due to the higher oxygen extraction in the upper body, the time trends of SmvO_2_ and ScvO_2_ are comparable under most circumstances^9–11^. While the measurement of SmvO_2_ requires pulmonary artery catheterization, ScvO_2_ can be more easily obtained from a CVC.

ScvO_2_ is determined by oxygen delivery to and oxygen consumption of the arms, head, and upper portion of the torso; the former depends on the arterial blood oxygen content and the cardiac output (CO). At rest with stable arterial oxygen saturation (SaO_2_), hemoglobin, and tissue oxygen consumption, ScvO_2_ can serve as a surrogate of CO. Poor oxygen delivery can be caused by decreased CO, e.g. from CHF or reduced cardiac preload, or decreased arterial oxygen content, e.g. due to anemia or hypoxemic states. Oxygen consumption is determined by metabolic status and is altered in sepsis, fever, exercise and sedation^12^. ScvO_2_ in the general population is poorly defined, as obtaining this measurement requires a CVC, and patients who require CVC placement are generally significantly ill. One study in healthy subjects found a ScvO_2_ of 76.8 ± 5.2% during cardiac catheterization^13^.

Studies in non-uremic populations, have found that abnormal ScvO_2_ levels are associated with worse morbidity and mortality^8, 14–18^. ScvO_2_ levels in HD patients have not been well described. In patients who have ESRD with CVC as vascular access, ScvO_2_ can be easily and continuously obtained during HD treatments by using the Crit-Line monitor^TM^ (CLM). The CLM is used routinely in Renal Research Institute HD units, which allowed us to investigate the ScvO_2_ in maintenance HD patients. The goals of our study were to evaluate the baseline characteristics of patients with different levels of intradialytic ScvO_2_ and to examine the associations between ScvO_2_ and mortality.

## Results

### Baseline patient characteristics

**Figure 1:**
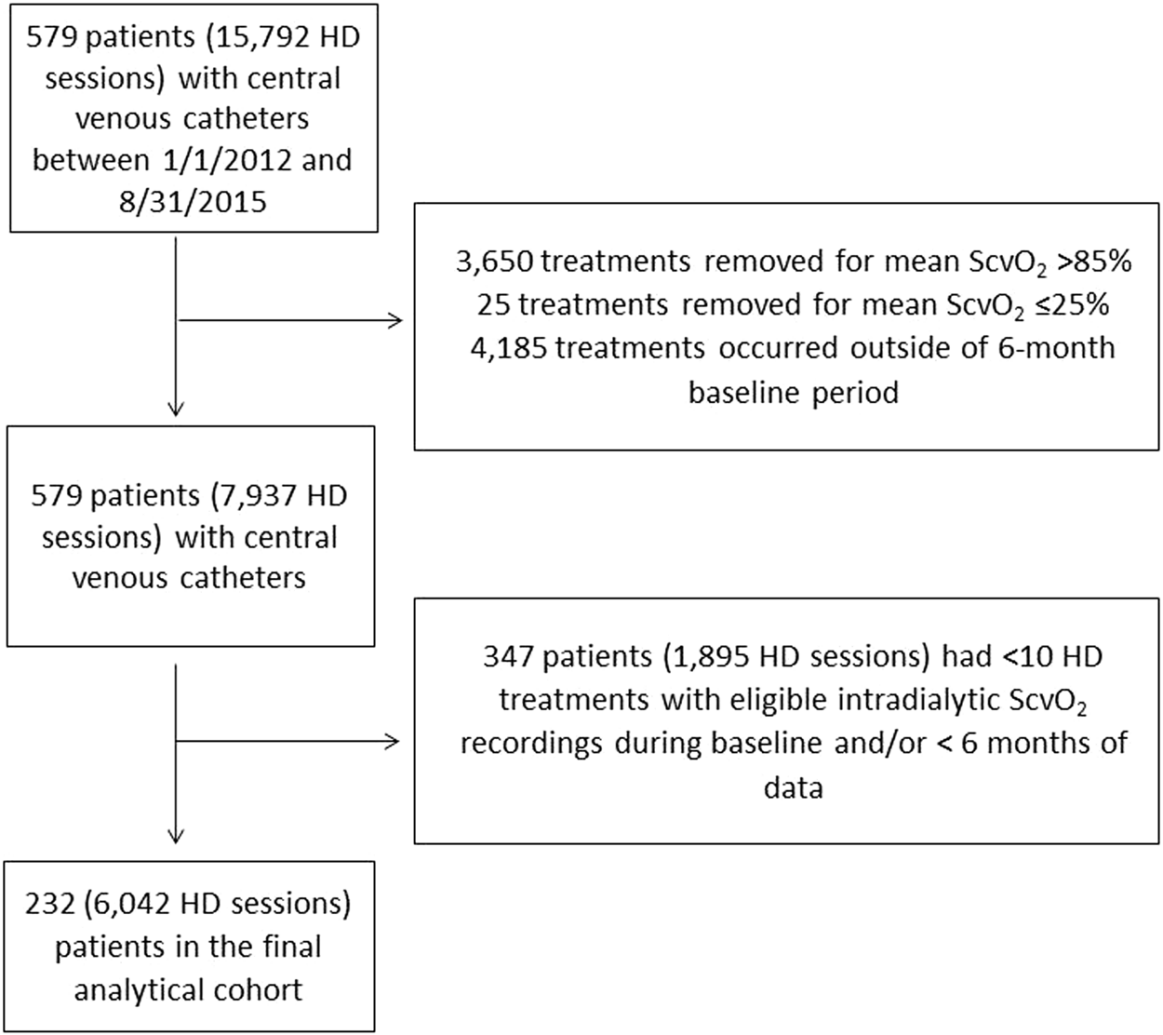
Study flow chart. HD: hemodialysis, ScvO_2_: central venous oxygen saturation.

The final analytical cohort comprised of 232 patients with 6,042 HD treatments and was derived after a deliberate step-by-step data cleaning process at the treatment level. Patients were only excluded in the event that they did not contribute sufficient data during baseline, either because of end of study, death, treatment modality change, recovery of renal function, or transfer to another dialysis facility (Fig. 1).

The initial population comprised of 579 patients with CVC as dialysis access, with a total of 15,792 HD treatments with ScvO_2_ measurements from January 1, 2012 until August 31, 2015. We excluded 3,650 treatments (23%) as they had a mean ScvO_2_ of greater than 85% and 25 treatments (0.16%) as they had a mean ScvO_2_ of less than or equal to 25%. We also excluded 4,185 treatments (26.5%) that occurred after the 6-month baseline period. This left us with 579 patients and 7,937 HD sessions, from which we excluded 347 patients with 1,895 HD treatments from the subsequent analysis because they had less than the required 10 HD treatments with ScvO_2_ recordings and/or less than 6 months of follow up **(**Fig. 1 **)**. Out of the 155 patients excluded for not having 6 months of follow up time, 79 were due to death.

In our study population, the mean age was 62.7 ± 15.7 years, dialysis vintage was 2.9 ± 4.6 years, 56% were white, 48.3% were male, 59% had diabetes mellitus (DM), 22% had CHF, and 10.3% had chronic obstructive pulmonary disease (COPD) **(**Table 1). Median follow-up time was 431 days.

**Table 1:** Baseline characteristics of all patients, lower tertiles and upper tertile.

Variables	All patients Mean ± SD	Lower Two Tertiles Mean ± SD	Upper Tertile Mean ± SD	Group Difference Mean (95% CI)	P- value
Patients [N]	232	154	78		
Number of eligible HD treatments during baseline [per patient]	26.1 ± 13.3	26.1 ± 13.1	26.1 ± 13.8	0.0 (−3.8 to 3.6)	0.953^a^
**Demographics**					
Age [years]	62.7 ± 15.7	66.0 ± 13.8	56.2 ± 17.3	9.8 (5.3 to 14.2)	0.001^a^
Race [% white]	56.0	53.9	60.3	−6.4	0.357^b^
Gender [% male]	48.3	48.1	48.7	−0.6	0.924^b^
Vintage [years]	2.9 ± 4.6	3.3 ± 5.1	2.0 ± 3.6	1.3	0.0136^c^
BMI [kg/m^2^]	28.1 ± 6.9	28.6 ± 7.0	27.3 ± 6.5	1.2 (−0.7 to 3.2)	0.207^a^
**ScvO**_**2**_ **saturation** [**%**]					
Mean ScvO_2_	58.7 ± 7.3	54.9 ± 5.3	66.3 ± 4.2	−11.4 (−12.6 to −10.1)	n.a.
Median ScvO_2_	59.1 ± 7.3	55.2 ± 5.3	66.6 ± 4.2	−11.4 (−12.7 to −10.1)	n.a.
Minimum ScvO_2_	48.4 ± 9.7	44.4 ± 8.9	56.3 ± 5.7	−11.9 (−13.8 to −10.0)	n.a.
Maximum ScvO_2_	65.2 ± 6.2	62.0 ± 4.6	71.5 ± 3.6	−9.5 (−10.6 to −8.4)	n.a.
SD ScvO_2_	3.4 ± 1.1	3.6 ± 1.1	2.9 ± 0.8	0.7 (0.4 to 0.9)	<0.001^a^
Start ScvO_2_	59.1 ± 7.4	55.4 ± 5.6	66.5 ± 4.4	−11.1 (−12.4 to −9.8)	n.a.
End ScvO_2_	57.3 ± 7.8	53.5 ± 6.2	64.8 ± 4.8	−11.3 (−12.7 to −9.8)	n.a.
End – Start ScvO_2_	−1.8 ± 3.6	−1.9 ± 3.7	−1.7 ± 3.5	−0.2 (−1.2 to 0.8)	0.62
**Comorbidities [%]**					
Diabetes	59.0	60.4	56.4	4.0	0.560^b^
CHF	22.0	21.4	23.1	−1.7	0.775^b^
COPD	10.3	11.0	9.0	2.0	0.626^b^
**Treatment parameters**					
Pre-dialysis SBP [mmHg]	146.4 ± 22.0	143.7 ± 22.9	151.7 ± 19.1	−8.0 (−14.0 to −2.1)	0.009^a^
Post-dialysis SBP [mmHg]	140.3 ± 20.1	137.8 ± 20.5	145.5 ± 18.2	−7.6 (−13.0 to −2.2)	0.006^a^
Peridialytic SBP change [mmHg]	−6.1 ± 11.9	−6.0 ± 11.7	−6.4 ± 12.4	0.4 (−2.9 to 3.7)	0.820^a^
IDWG [kg]	2.0 ± 0.8	1.9 ± 0.8	2.1 ± 0.8	−0.12 (−0.3 to 0.1)	0.249^a^
IDWG relative to post-dialysis weight [%]	2.6 ± 0.9	2.5 ± 0.9	2.8 ± 1.0	−0.3 (−0.6 to −0.1)	0.007^a^
UFV [L]	1.9 ± 0.8	1.9 ± 0.79	2.0 ± 0.8	−0.1 (−0.4 to 0.1)	0.173^a^
Normalized UFV [mL/kg]	25.3 ± 9.7	24 ± 8.9	28 ± 10.7	−4 (−6.6 to −1.4)	0.003^a^
Post-dialysis weight [kg]	77.4 ± 20.4	79.0 ± 21.4	74.3 ± 18.1	4.6 (−0.9 to 10.2)	0.102^a^
Treatment time [minutes]	219.0 ± 23	217.7 ± 23.8	221.5 ± 21.1	−3.7 (−10.1 to 2.5)	0.235^a^
Equilibrated Kt/V	1.5 ± 0.3	1.5 ± 0.3	1.5 ± 0.2	0.0 (−0.1 to 0.1)	0.610^a^
**Laboratory parameters**					
Hgb [g/dL]	10.6 ± 0.9	10.6 ± 0.9	10.6 ± 0.96	0.0 (−0.3 to 0.3)	0.962^a^
Serum sodium [mmol/L]	138.6 ± 3.1	138.6 ± 3.2	138.7 ± 2.8	−0.1 (−0.9 to 0.7)	0.782^a^
Serum potassium [mmol/L]	4.7 ± 0.6	4.6 ± 0.6	4.7 ± 0.4	−0.1 (−0.2 to 0.1)	0.292^a^
Intact PTH [pg/mL]	518.3 ± 481.1	538.3 ± 498.3	478.6 ± 445.4	59.7 (−72.9 to 192.2)	0.376^a^
Serum bicarbonate [mmol/L]	23.4 ± 2.2	23.3 ± 2.3	23.7 ± 2.2	−0.4 (−1.0 to 0.2)	0.165^a^
Leukocytes [1000/µL]	7.0 ± 2.0	7.2 ± 2.1	6.6 ± 1.7	0.6 (0.1 to 1.1)	0.019^a^
Platelets [1000/µL]	212.9 ± 63.9	216.9 ± 65.1	205.1 ± 61.3	11.8 (−6.5 to 30.1)	0.204^a^
NLR	4.4 ± 2.6	4.6 ± 2.8	3.8 ± 2.0	0.79 (0.2 to 1.4)	0.015^a^
Serum albumin [g/dL]	3.8 ± 0.4	3.7 ± 0.4	3.8 ± 0.4	−0.1 (−0.2 to 0.04)	0.165^a^
Ferritin [ng/mL]	780.5 ± 510.2	798.8 ± 487.9	744.7 ± 552.9	54.1 (−86.8 to 195.0)	0.45^a^
Transferrin saturation [%]	30.7 ± 9.5	29.8 ± 8.9	32.6 ± 10.5	−2.8 (−5.4 to −0.2)	0.036^a^

95% CI, 95% confidence interval; SD, standard deviation; ScvO_2_, central venous oxygen saturation; BMI, body mass index; CHF, congestive heart failure; COPD, chronic obstructive pulmonary disease; SBP, systolic blood pressure; UFV, ultrafiltration volume; IDWG, interdialytic weight gain; Hgb, hemoglobin; PTH, parathyroid hormone; NLR, neutrophil-to-lymphocyte ratio; n.a., not applicable.

^a^ *t* test.

^b^Chi-square test.

^c^Wilcoxon test.

**Figure 2:**
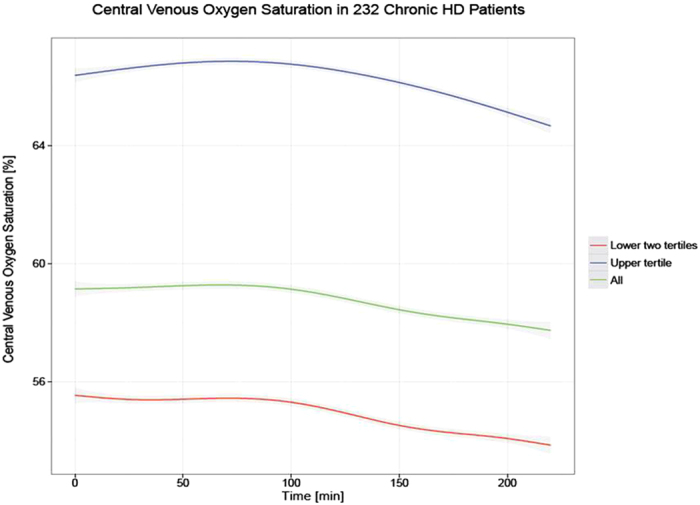
Time course of mean ScvO_2_ during hemodialysis in all patients (green), lower two tertiles (red) and upper tertile (blue). The respective 95% confidence intervals are indicated in gray.

During baseline, ScvO_2_ was recorded in 26 ± 13.3 HD treatments per patient. On a population level the ScvO_2_ was normally distributed with a mean of 58.7 ± 7.3%. Analysis of intradialytic ScvO_2_ dynamics across all patients indicated that on average ScvO_2_ slightly increased over the first 60 minutes of treatment, and then progressively declined below starting levels towards the end of HD (Fig. 2).

### ScvO_2_ as a dichotomous outcomes

#### Comparison of baseline characteristics between upper and lower tertiles

Patients were stratified into upper tertile (N = 78) and lower two tertiles (N = 154); a mean ScvO_2_ level below 61.1% during baseline period separated the two groups. A comparison of baseline characteristics between upper and lower tertiles is presented in Table 1. The patients in the lower tertiles were older (66.0 ± 13.8 years vs 56.2 ± 17.3 years, P < 0.001), had longer dialysis vintage (3.3 ± 5.1 years vs 2.0 ± 3.6 years, P = 0.031), lower pre-dialysis systolic blood pressure (SBP) (143.7 ± 22.9 mmHg vs 151.7 ± 19 mmHg, P = 0.009), lower post-dialysis SBP (137.8 ± 20.5 mmHg vs 145.5 ± 18.2 mmHg, P = 0.006), and had lower normalized UFR (6.8 ± 2.4 mL/kg/hr vs 7.7 ± 2.9 mL/kg/hr, P = 0.015). Furthermore, lower tertile subjects had higher leukocyte counts (7.2 ± 2.1 * 1000/µL vs 6.6 ± 1.7 * 1000/µL, P = 0.019) and higher neutrophil-to-lymphocyte ratios (NLR) (4.6 ± 2.8 vs 3.8 ± 2.0, P = 0.015). There was no statistically significant difference in comorbidities of DM, CHF or COPD.

#### Mortality between upper and lower tertiles

**Figure 3:**
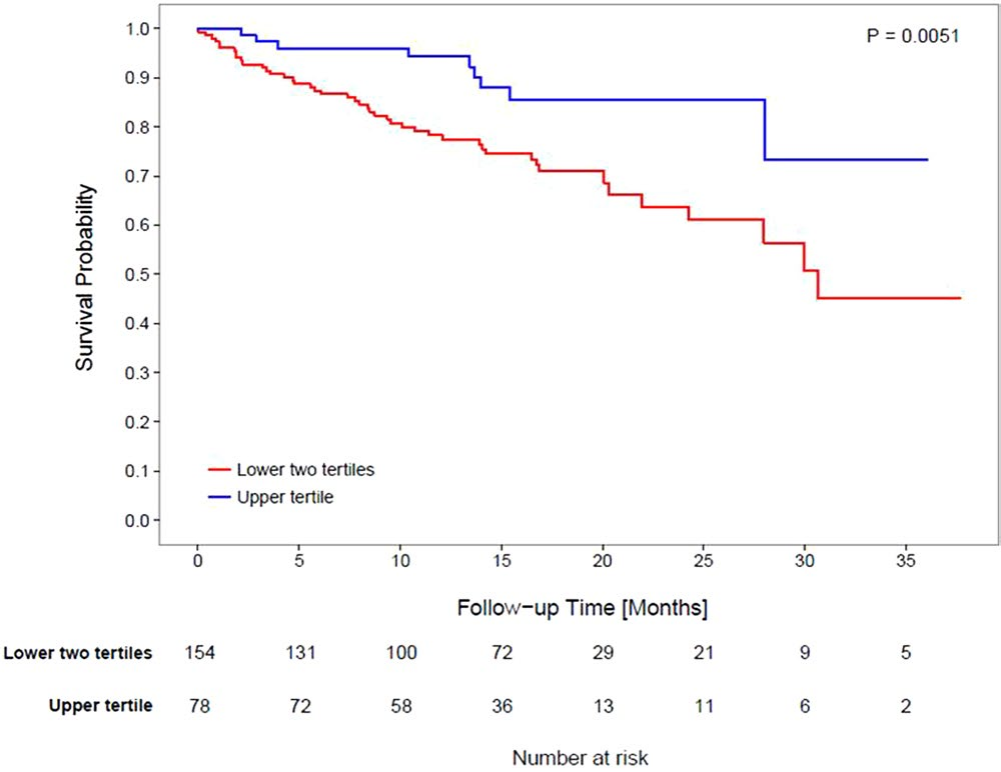
Kaplan-Meier estimates for survival probabilities in the lower two tertiles (red) and the upper tertile (blue), respectively. Median follow up for the lower two tertiles was 428 days while the median follow up time for the upper tertile was 432 days. The number of patients at risk is indicated in the table below the graph. The time to death differs significantly between the two groups (P = 0.0051, log-rank test).

During the 36-month follow-up period, there were a total of 54 deaths, 45 in the lower two tertiles and 9 in the upper tertile. Mortality rate was 24.1/100 patient years in lower two tertiles and 9.0/100 patient years in upper tertile (P = 0.005). Univariate Kaplan-Meier analysis indicated a significantly shorter survival among lower tertile patients (P = 0.0051, log-rank test) (Fig. 3).

### ScvO_2_ as a continuous variable

In unadjusted Cox analysis, for every 1 percent point decrease in mean ScvO_2_ there was an associated 6% increase in mortality (HR 1.06 (1.03–1.10)). There was no material change in the results after adjustment for age, gender, comorbidities (COPD and CHF), log vintage, inflammatory markers (albumin, NLR), hemoglobin and erythropoietin dose (HR 1.04 (1.01–1.08)) **(**Table 2 **)**.

**Table 2:** Crude and adjusted hazard ratios for all-cause mortality for a 1% decrease in central venous oxygen saturation.

Outcome	Events	Crude^a^		Minimally Adjusted^b^		Fully Adjusted^c^	
		HR (95% CI)	P Value	HR (95% CI)	P Value	HR (95% CI)	P Value
All-cause mortality	54	1.06 (1.03 to 1.10)	<0.001	1.05 (1.02 to 1.09)	0.003	1.04 (1.01 to 1.08)	0.0437

HR, hazard ratio.

^a^Unadjusted model.

^b^Adjusted for age, gender, chronic obstructive pulmonary disease and congestive heart failure.

^c^Adjusted for age, gender, chronic obstructive pulmonary disease, congestive heart failure, albumin, hemoglobin, erythropoietin dose, neutrophil to lymphocyte ratio, and log vintage.

#### Correlates of ScvO_2_

**Figure 4:**
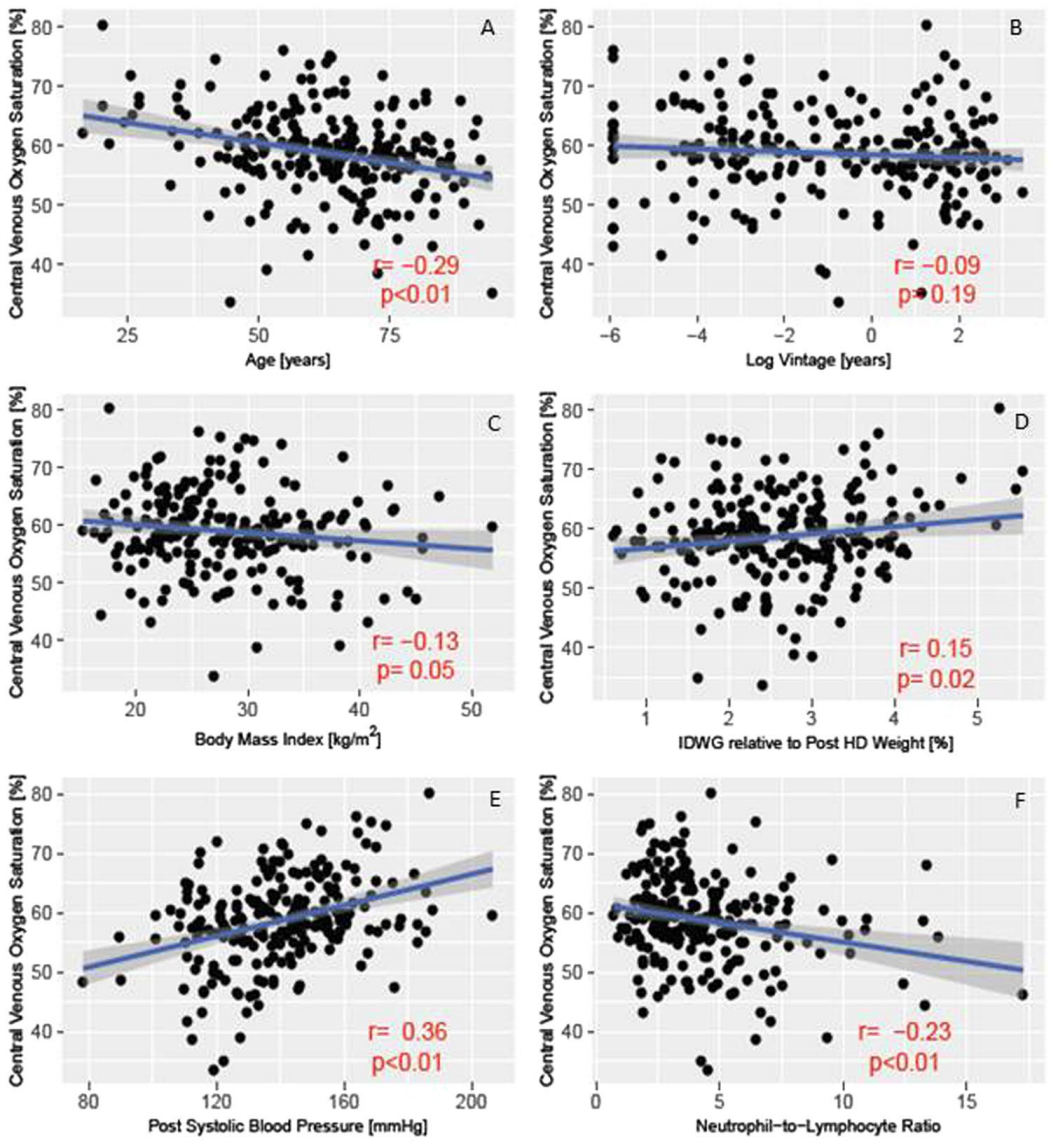
Correlates of central venous oxygen saturation with respect to patient characteristics. Each point represents one patient; the depicted data points represent the respective parameter averages during the 6-month baseline period. (**A**) Age; (**B**) Log vintage; (**C**) Body mass index; (**D**) Interdialytic weight gain relative to post-dialysis body weight; (**E**) Post-dialysis systolic blood pressure; (**F**) Neutrophil-to-lymphocyte ratio.

Figure 4 depicts the relationship between ScvO_2_ and patient characteristics that were found to differ between the two groups. Mean ScvO_2_ across patients was plotted against age, log vintage, body mass index (BMI), interdialytic weight gain (IDWG) relative to post-HD weight, post-HD SBP, and NLR. As vintage was not normally distributed, it was log transformed. Age, BMI, log vintage and NLR were negatively associated with ScvO_2_, while post-HD SBP and IDWG were positively correlated with ScvO_2_. While all correlates were statistically significant except for ScvO_2_ and log vintage (P = 0.19), correlation coefficients were relatively low.

## Discussion

Our study indicates that in chronic HD patients with CVC as vascular access, lower ScvO_2_ levels are associated with poorer survival.

Despite the relative ease with which ScvO_2_ can be obtained in HD patients with CVC as access, to date only small studies have examined this key indicator of cardiac function. Cordtz *et al*.^19^ in 2008 evaluated 20 HD patients and classified them as either hypotension prone or hypotension resistant and measured their ScvO_2_ at treatment initiation and end. The authors found a significant decrease in ScvO_2_ in hypotension prone patients. Harrison *et al*.^20^ investigated 18 HD patients and found a strong inverse correlation between ScvO_2_ at the end of dialysis and ultrafiltration volume normalized to post-HD body weight. A recent review of intradialytic oxygen saturation did not identify any previous research examining the association between ScvO_2_ and patient survival^21^.

In the study by Harrison *et al*. the mean ScvO_2_ was 63.5 ± 13% pre-HD and 56.4 ± 8% post-HD^20^, whereas in the study by Cordtz *et al*. the initial ScvO_2_ was 52.2 ± 6.7% in hypotension prone and 49.7 ± 6.9% in hypotension resistant patients^19^. While these studies focused on ScvO_2_ at HD start and end, we examined ScvO_2_ continuously throughout the HD session. The ScvO_2_ levels found in our study are below the levels of ~70% observed in healthy subjects^13^, but consistent with those reported in HD patients. The exact etiology of low intradialytic ScvO_2_ in HD patients is not well established, but may be partially explained by the lower hemoglobin levels, and the higher prevalence of cardiac dysfunction and pulmonary hypertension in HD patients.

In our cohort, when ScvO_2_ was assessed throughout the entire HD treatment, on average ScvO_2_ increased slightly over the first hour and then progressively declined during the remaining treatment time. The determinants of ScvO_2_ can be visualized by rearrangement of the familiar form of Fick’s law and replacement of SmvO_2_ with ScvO_2_, and CO with upper body blood flow (UBBF), which results in the following equation

1 $${\rm{ScvO}}2\,=\mathrm{SaO}2-\frac{100\ast {\rm{oxygen}}\,{\rm{consumption}}}{{\rm{K}}\ast {\rm{Hgb}}\ast {\rm{UBBF}}}$$

with ScvO_2_ and SaO_2_ in %, upper body oxygen consumption in mL/min, Hgb in g/L, UBBF in L/min, and K being 1.34, the amount of oxygen (in mL) bound per g of hemoglobin.

Therefore, there are four components which may change during HD and that will cause a decrease in ScvO_2_; (i) increased tissue oxygen consumption; (ii) a decrease in SaO_2_, (iii) a decrease in hemoglobin concentration, and (iv) a decrease in upper body blood flow. An increase in oxygen consumption can occur due to an increase in metabolic rate. A small study done in maintenance HD patients found that whole body energy expenditure, measured by indirect calorimetry, increased during HD^22^. However, to what extent the upper body energy expenditure changes during HD is currently unknown. It would be of interest to know if oxygen consumption by the brain, by far the largest consumer of oxygen in the upper body, changes during HD. Intradialytic SaO_2_ has been demonstrated to decrease during the first hour of HD; unfortunately we do not have SaO_2_ levels in our patients^23^. While HD patients have lower hemoglobin levels than the general population, during HD as UF occurs and the relative blood volume decreases, hemoglobin levels generally rise due to hemoconcentration^24^. We suspect that a reduction in CO and consequently a decrease in upper body blood flow is the predominant driving factor leading to a drop in ScvO_2_. The almost linear relation between cerebral perfusion and CO has been recently reviewed^25^. When faced with any of the other possibilities in a patient with intact cardiac function, there should be a compensatory response in CO^26^. There is growing literature on depressed CO during HD treatment due to poor vascular refill and regional wall motion abnormalities (RWMA)^27, 28^. In fact, a recent study using intradialytic magnetic resonance imaging of the heart demonstrated that systolic contractile function fell during HD, with all 12 patients experiencing some degree of segmental left ventricular dysfunction along with evidence of decreased intravascular volume and an inadequate heart rate response^29^.

ScvO_2_ in patients with sepsis, post-surgery, and trauma has been examined, with studies finding that abnormal ScvO_2_ levels are associated with increased morbidity and mortality^15, 16, 18^. However, the ESRD population is unique in many aspects, and the results of prior studies in other populations therefore may not be fully applicable.

We and others have observed a left shift of the ScvO_2_ distribution in HD patients compared to healthy subjects, possibly related to anemia and lower CO. Therefore we refrained from defining comparison groups based on ScvO_2_ levels obtained in healthy subjects but rather used ScvO_2_ tertiles obtained from our large HD population, where a ScvO_2_ of 61.1% separated the top from the bottom two tertiles^13, 30^. In a review of literature, ScvO_2_ levels below 64.4% were associated with morbidity post-surgery, and values below 62% were associated with mortality in patients with pulmonary hypertension^18, 31^. In the trauma setting, ScvO_2_ < 65% on initial evaluation in the emergency room predicted higher blood loss and greater severity of injuries^16^. We complemented this binary analysis with a continuous spline analysis of the association between ScvO_2_ and hazard ratio for all-cause mortality; that analysis indicated that mean ScvO_2_ levels below 63% were associated with increased mortality.

Our finding that patients with lower ScvO_2_ were older may reflect the poorer cardiac function expected in older subjects. Of note, the prevalence of CHF increases with age, as does CHF mortality^32^. On univariate analysis, age was an independent risk factor for mortality. However, even after adjustment for age in our analysis, ScvO_2_ as a continuous variable remained a significant predictor of mortality.

While the correlation coefficients were low, we identified several significant correlations between ScvO_2_ and patient variables such as the association between lower ScvO_2_ levels and longer dialysis vintage. We speculate that this finding may be related to recurrent hemodynamic stress and cardiac injury caused by HD. McIntyre *et al*. demonstrated that HD induced RWMA in a subset of maintenance HD patients. While at baseline there was no difference in left ventricular ejection fraction (LVEF) between patients who developed RWMA and those that did not, at 1 year follow-up, the group of patients that developed RWMA during HD had significantly lower resting LVEF^5^.

In our study, lower tertile patients had lower pre-HD and lower post-HD SBP, a finding possibly related to low CO. Of note, an association between low pre-HD SBP and mortality has been repeatedly shown^33, 34^. It is interesting to note that in our study the prevalence of CHF did not differ between lower and upper tertiles. Unfortunately, no routine echocardiography assessments were available in our patients, so we cannot comment on the possibility of deficient documentation, classification or misdiagnosis of CHF. One intriguing possibility is that we may be identifying a group of patients without clinically overt signs and symptoms of CHF at rest, who however have reduced cardiac reserve or autonomic dysfunction and are unable to mount the necessary increase in sympathetic response and CO when faced with the hemodynamic stress of HD^27, 35^.

The main limitation of our study is its observational nature, which prevents any conclusions related to causality. As mentioned earlier, routine echocardiograms are unfortunately not available in our study population, making potentially very insightful correlational analyses of ScvO_2_ and cardiac structure and function impossible. Lastly, we appreciate that ScvO_2_ measurements may be altered by changes in catheter tip position due to changes in body position; however, we have no indication that this may affect one of the two groups disproportionally and created any bias.

Considering a recent review of this topic, we believe that this is the largest study to date examining the epidemiology of ScvO_2_ in maintenance HD patients^21^. CVC are used as vascular access in the majority of U.S. patients starting HD^1^. While this situation is certainly not desirable, the presence of a CVC allows us to measure ScvO_2_, a vitally important physiological parameter. This additional diagnostic opportunity may be particularly important in the incident period, the time with the highest cardiovascular morality rate^1^. In fact, a recent study published by Mancini *et al*. demonstrates that variability in SaO_2_ is associated with intradialytic hypotension^36^. This supports the potential role of oxygen saturation monitoring during dialysis.

In conclusion, our research shows that routine measurement of ScvO_2_ during HD provides a novel window into patients’ biology that may help to improve our care for this vulnerable patient population.

## Method

### Population and study design

**Figure 5:**
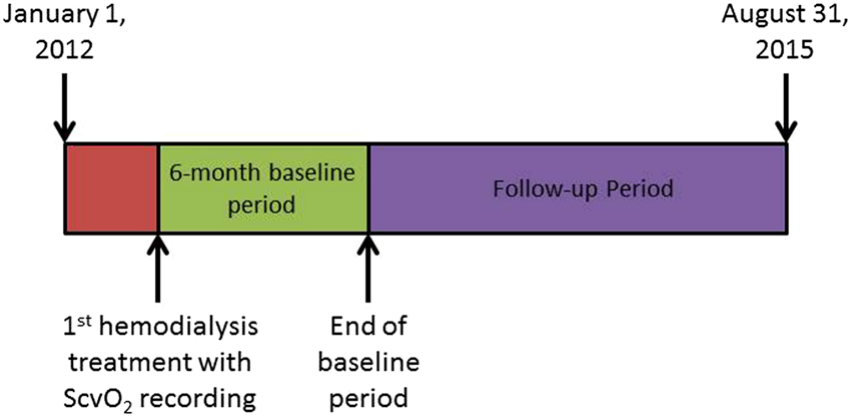
Data were reviewed starting from January 1, 2012. Due to the staggered deployment of Crit-line monitors to dialysis units, patients were enrolled into the study on a rolling basis. The first hemodialysis treatment with ScvO_2_ measurements marked the beginning the 6-month baseline period. Follow-up ended with either end of study (August 31, 2015), death, treatment modality change, recovery of renal function, or transfer to another dialysis facility. ScvO_2_: central venous oxygen saturation.

This is a retrospective multi-center study of a cohort of maintenance HD patients from 17 facilities of the Renal Research Institute (RRI) across the United States between January 2012 and August 2015. In these clinics, CLM use is part of standard care. All patients were treated with bicarbonate dialysate and polysulfone membranes. Over 80% of patients had a prescribed dialysate temperature of 37 °C. All patients who received HD via a CVC and had at least 6 months of clinical data and 10 dialysis treatments with eligible ScvO_2_ recordings (definition of eligibility see below) were eligible for inclusion into the study. Therefore our study included both incident and prevalent HD patients. The CLM was rolled out into dialysis units in a staggered manner, and we used the first treatment with CLM data as start date of the patients’ 6 month baseline period. Since eligible patients had to contribute 6 months’ worth of data, by design only those patients who survived for at least 6 months were included into the study (Fig. 1). Patient characteristics were assessed over the baseline period, and mortality was assessed during a follow-up period for a maximum duration of three years. Figure 5 summarizes the study design. For group comparison patients were stratified based on the population ScvO_2_ that separated the top tertile from the bottom two tertiles. Descriptive statistics of the ScvO_2_ distribution showed a ScvO_2_ of 61.1% to be the cut-off between these two groups. Patients were censored in the event of kidney transplantation, transfer to a non-RRI facility, dialysis treatment modality change, recovery of kidney function, or end of follow-up.

The study was approved by the New England Institutional Review Board (14–446) and conducted in accordance with the Declaration of Helsinki. Informed consent was not obtained as this was determined not to be human subject research, and we were working with de-identified data.

This study has been registered at clinicaltrials.gov (NCT02501044).

### Measurement of ScvO_2_

Intradialytic ScvO_2_ measurements were obtained by the CLM. The CLM has been approved by the U.S. Food and Drug Administration (FDA) for the measurement of hematocrit, relative blood volume, and oxygen saturation in the extracorporeal dialysis circuit. The CLM measures oxygen saturation 9,000 times per minute and reports the mean of these measurements every minute. The manufacturer reported accuracy for oxygen saturation measurement is 2%. Patients’ mean, median, minimum, maximum, standard deviation, start-HD, end-HD ScvO_2_ was calculated per treatment and then averaged across all treatments per patient and subsequently across patients. We chose to do our analysis using the mean ScvO_2_ as there was low variability across treatments for each patient (mean coefficient of variability of 7.5 ± 4%).

### Clinical and laboratory data

Laboratory measurements were done at Spectra East Laboratories (Rockleigh, NJ, USA). The results were downloaded to the RRI data warehouse and extracted to the study database. Continuous variables were averaged during the baseline period. BMI was calculated using post-HD dry weight.

### Data eligibility

To ensure appropriate data quality, we included only treatments where mean ScvO_2_ was below 85%, as higher values are incompatible with central venous blood^13^. Mean ScvO_2_ measurements less than 25% were excluded because they are considered incompatible with life^37^. Additionally, data points with relative blood volume measurements above 102% were considered very unlikely, potentially due to saline administration, and hence excluded. This constituted 3% of all data points.

### Comorbidities

CHF, DM, and COPD were defined using International Classification of Diseases - 9 (ICD-9) codes.

### Statistical analysis

Continuous variables are presented as mean ± standard deviation (SD) if normally distributed and as median (25^th^, 75^th^ percentile) otherwise. Categorical variables are presented as percentages of the respective group. Statistics of ScvO_2_ variables were calculated on a HD treatment level and then aggregated on a patient level.

Baseline characteristics of exposed and unexposed were compared using chi-square test for categorical variables and two-sample *t* test for continuous variables, Wilcoxon Rank-Sum test were used for non-parametric variables. Survival characteristics were compared using Kaplan-Meier plots, log-rank test, and Cox proportional hazards models.

Statistical analyses were performed using SAS version 9.3 (SAS Institute Inc., Cary, NC) and R 3.0.2 (libraries ggplot2, splines, survival, pspline; R Foundation for Statistical Computing, Vienna, Austria).

### Disclosure

P.K. holds stock in Fresenius Medical Care. L.C. is supported in part by the NIH (5T32DK007757-18). The other authors declared no competing interest. The results presented in this paper have not been published previously in whole or part, except in abstract format.

### Data Availability

Consolidated data may be shared with other scientists at their request.

## References

[1] United States Renal Data System. 2015 USRDS annual data report: Epidemiology of kidney disease in the United States. (National Institutes of Health, National Institute of Diabetes and Digestive and Kidney Diseases, Bethesda, MD, 2015).

[2] Ragosta M (2004). Coronary flow reserve abnormalities in patients with diabetes mellitus who have end-stage renal disease and normal epicardial coronary arteries. American heart journal.

[3] Schneider A (2013). Determinants of cardiovascular risk in haemodialysis patients: post hoc analyses of the AURORA study. Am J Nephrol.

[4] Stefansson BV (2014). Intradialytic hypotension and risk of cardiovascular disease. Clinical journal of the American Society of Nephrology: CJASN.

[5] Burton JO, Jefferies HJ, Selby NM, McIntyre CW (2009). Hemodialysis-induced cardiac injury: determinants and associated outcomes. Clinical journal of the American Society of Nephrology: CJASN.

[6] Zoccali C, Mallamaci F, Tripepi G (2002). Nocturnal hypoxemia predicts incident cardiovascular complications in dialysis patients. Journal of the American Society of Nephrology: JASN.

[7] Zoccali C (1998). Nocturnal hypoxemia, night-day arterial pressure changes and left ventricular geometry in dialysis patients. Kidney international.

[8] Rivers E (2001). Early goal-directed therapy in the treatment of severe sepsis and septic shock. The New England journal of medicine.

[9] Schou H, Perez de Sa V, Larsson A (1998). Central and mixed venous blood oxygen correlate well during acute normovolemic hemodilution in anesthetized pigs. Acta anaesthesiologica Scandinavica.

[10] Chawla LS (2004). Lack of equivalence between central and mixed venous oxygen saturation. Chest.

[11] Scheinman MM, Brown MA, Rapaport E (1969). Critical assessment of use of central venous oxygen saturation as a mirror of mixed venous oxygen in severely ill cardiac patients. Circulation.

[12] Vincent, J. L. *Ebrary Inc. Yearbook of intensive care and emergency medicine* (Springer, Berlin, 2005).

[13] Barratt-Boyes BG, Wood EH (1957). The oxygen saturation of blood in the venae cavae, right-heart chambers, and pulmonary vessels of healthy subjects. The Journal of laboratory and clinical medicine.

[14] Balzer F (2015). High central venous saturation after cardiac surgery is associated with increased organ failure and long-term mortality: an observational cross-sectional study. Critical care.

[15] Textoris J (2011). High central venous oxygen saturation in the latter stages of septic shock is associated with increased mortality. Critical care.

[16] Scalea TM (1990). Central venous oxygen saturation: a useful clinical tool in trauma patients. The Journal of trauma.

[17] Futier E (2010). Central venous O(2) saturation and venous-to-arterial CO(2) difference as complementary tools for goal-directed therapy during high-risk surgery. Critical care.

[18] Pearse R (2005). Changes in central venous saturation after major surgery, and association with outcome. Crit Care.

[19] Cordtz J, Olde B, Solem K, Ladefoged SD (2008). Central venous oxygen saturation and thoracic admittance during dialysis: new approaches to hemodynamic monitoring. Hemodial Int.

[20] Harrison LE, Selby NM, McIntyre CW (2014). Central venous oxygen saturation: a potential new marker for circulatory stress in haemodialysis patients?. Nephron. Clinical practice.

[21] Campos I (2016). Intradialytic Hypoxemia in Chronic Hemodialysis Patients. Blood purification.

[22] Ikizler TA (1996). Increased energy expenditure in hemodialysis patients. Journal of the American Society of Nephrology: JASN.

[23] Meyring-Wösten A (2016). Intradialytic Hypoxemia and Clinical Outcomes in Patients on Hemodialysis. Clinical Journal of the American Society of Nephrology.

[24] Wink J, Vaziri ND, Barker S, Hyatt J, Ritchie C (1988). The effect of hemodialysis on whole blood, plasma and erythrocyte viscosity. The International journal of artificial organs.

[25] Meng L, Hou W, Chui J, Han R, Gelb AW (2015). Cardiac Output and Cerebral Blood Flow: The Integrated Regulation of Brain Perfusion in Adult Humans. Anesthesiology.

[26] Blumberg A, Keller G (1979). Oxygen consumption during maintenance hemodialysis. Nephron.

[27] Daugirdas JT (1991). Dialysis hypotension: a hemodynamic analysis. Kidney international.

[28] Selby NM, Lambie SH, Camici PG, Baker CS, McIntyre CW (2006). Occurrence of regional left ventricular dysfunction in patients undergoing standard and biofeedback dialysis. American journal of kidney diseases: the official journal of the National Kidney Foundation.

[29] Buchanan, C. *et al*. Intradialytic Cardiac Magnetic Resonance Imaging to Assess Cardiovascular Responses in a Short-Term Trial of Hemodiafiltration and Hemodialysis. *Journal of the American Society of Nephrology* (2016). 10.1681/ASN.2016060686 PMC5373461 28122851

[30] Madsen P, Iversen H, Secher NH (1993). Central venous oxygen saturation during hypovolaemic shock in humans. Scandinavian journal of clinical and laboratory investigation.

[31] Sitbon O (2002). Long-term intravenous epoprostenol infusion in primary pulmonary hypertension: prognostic factors and survival. Journal of the American College of Cardiology.

[32] Schocken DD, Arrieta MI, Leaverton PE, Ross EA (1992). Prevalence and mortality rate of congestive heart failure in the United States. Journal of the American College of Cardiology.

[33] Li Z (2006). The epidemiology of systolic blood pressure and death risk in hemodialysis patients. American journal of kidney diseases: the official journal of the National Kidney Foundation.

[34] Port FK (1999). Predialysis blood pressure and mortality risk in a national sample of maintenance hemodialysis patients. American journal of kidney diseases: the official journal of the National Kidney Foundation.

[35] Chesterton LJ (2010). Categorization of the hemodynamic response to hemodialysis: the importance of baroreflex sensitivity. *Hemodialysis international*. International Symposium on Home Hemodialysis.

[36] Mancini, E. *et al*. Intra-dialytic blood oxygen saturation (SO2): association with dialysis hypotension (the SOGLIA Study). *Journal of nephrology* (2016). 10.1007/s40620-016-0346-x 27572624

[37] Marx G, Reinhart K (2006). Venous oximetry. Current opinion in critical care.

